# Endovascular intravascular intervention for central venous stenosis in maintenance hemodialysis patients: a retrospective observational study

**DOI:** 10.3389/fcvm.2024.1405606

**Published:** 2024-12-09

**Authors:** Yiming Tao, Jianyun Wang, Jianchao Ma, Siqi Peng, Boxi Chen, Shuting Deng, Ye Yuan, Nan Jiang, Sichun Wen, Bohou Li, Qiong Wu, Lixia Xu, Sijia Li, Ting Lin, Feng Wen, Lei Fu, Zhuo Li, Renwei Huang, Chaosheng He, Wenjian Wang, Zhiming Ye, Wei Shi, Zhonglin Feng, Shuangxin Liu

**Affiliations:** ^1^ Department of Nephrology, Guangdong Provincial People's Hospital (Guangdong Academy of Medical Sciences), Southern Medical University, Guangzhou, China; ^2^ Department of Nephrology, Gaozhou People’s Hospital, Gaozhou, China

**Keywords:** hemodialysis, central venous stenosis, percutaneous transluminal angioplasty, percutaneous transluminal stenting, patency rates

## Abstract

**Background:**

The number of people undergoing maintenance hemodialysis is increasing rapidly worldwide. Central vein stenosis (CVS) is a common vascular complication in undergoing hemodialysis, especially those with a history of catheterization. This study aimed to investigate the characteristics of CVS and the clinical effectiveness of percutaneous transluminal angioplasty (PTA) alone and sequential percutaneous transluminal stenting (PTS) in hemodialysis patients with CVS.

**Methods:**

A retrospective analysis of 26 cases of endovascular intervention for CVS using PTA alone or sequential PTS was performed. The characteristics of CVS and the clinical effectiveness of these procedures were evaluated.

**Results:**

This study included 26 hemodialysis patients who presented with symptomatic CVS. Of these 26 patients, 53.85% were male, and their mean age was approximately 54.96 years. All the patients had a history of catheter placement or pacemaker implantation. The incidence of brachiocephalic vein stenosis was significantly higher than that of subclavian vein stenosis (46.16% vs. 26.92%). Based on the degree of stenosis and elastic retraction, these patients were administered PTA alone or sequential PTS. There was no difference in patient age, hemodialysis time, catheter retention time, or stenosis length between the PTA alone and sequential PTS groups. However, the degree of venous stenosis in the PTS group was more severe than that in the PTA alone group. The primary patency rates in the sequential PTS and PTA alone groups were 94.12% and 100% at 3 months; 88.24% and 88.89% at 6 months; 75.00% and 85.71% at 9 months; and 66.67% and 71.43% at 12 months, respectively. It is worth noting that for 7 patients with complete occlusion of the brachiocephalic vein, we used sharp recanalization technology and stenting placement, with patency rates of 85.71% and 71.43% at 6 and 12 months, respectively.

**Conclusions:**

PTA alone is recommended for patients with less than 50% central venous elastic retraction, while sequential PTS is recommended for patients with ≥ 50% central venous elastic retraction. PTA and PTS are safe and effective methods for the treatment of CVS in patients undergoing hemodialysis.

## Introduction

Over the past 30 years, the number of patients on dialysis has increased significantly worldwide. It is estimated that there were more than 2 million dialysis patients worldwide in 2010, and modelling data show that this number will more than double by 2030 (1, 2). Although there has been a significant increase in hemodialysis patients, most of the patients at the initiation of hemodialysis need to be placed on a non-tunneled catheter in developed or developing countries (3–5). The tunneled catheter can be used as a long-term vascular access for dialysis patients. However, tunneled catheter often stays longer than recommended, and catheter indwelling time is associated with the occurrence of central vein stenosis (1, 2). Due to catheterization at hemodialysis initiation, there are more clinical problems associated with vascular access in patients undergoing hemodialysis. Central vein stenosis (CVS) is a hemodialysis access-related complication (6). Although hemodialysis patients with CVS are asymptomatic, the clinical picture of the symptomatic lesion is quite typical if the lesion is ipsilateral to the arteriovenous hemodialysis access. Symptomatic patients characteristically develop ipsilateral arm edema, which is often progressive and can become severe. Severe extremity edema can cause considerable patient discomfort and increase the risk of serious complications such as skin ulceration and infection. Typically, swelling of the ipsilateral shoulder, breast, neck, and face develops as edema of the arm progresses (7, 8).

The treatment of CVS is aimed at improving the symptoms of venous stenosis and providing the vascular access needed for adequate dialysis. Patients requiring treatment have venous stenosis with a reduced lumen diameter of >50% and clinical/physiologic abnormalities (9, 10). According to the KDOQI Clinical Practice Guideline for Vascular Access:2019 Update, endovascular interventions are preferred for CVS (11). However, the clinical efficacy of this treatment has been less reported. We retrospectively investigated the clinical outcomes of 26 patients treated with percutaneous transluminal angioplasty (PTA) alone or sequential percutaneous transluminal stenting (PTS).

## Method

### Study subjects

Maintenance hemodialysis patients who received interventional treatment for CVS at Guangdong Provincial People's Hospital between November 2018 and November 2021 were retrospectively selected for this study. The inclusion criteria were as follows: (I) 18 years of age or older, (II) maintenance hemodialysis, (III) CVS was confirmed through enhanced CT, (IV) CVS with a reduced lumen diameter of >50%, and (V) central venous combined with limb edema or hemodialysis vascular access dysfunction. The exclusion criteria were as follows: (I) CVS with a reduced lumen diameter of <50%, (II) no clinical/physiologic abnormalities, and (III) ipsilateral vein with a central venous catheter (12, 13). A total of 27 eligible patients were enrolled in the study, of whom 1 patient refused to provide follow-up data and was excluded.

### Endovascular treatment technique

All procedures were preceded by the application of PTA for predilatation, and subsequent additional stent placement was selected based on clinical judgment (14). All enrolled patients were first treated with central venography, no patients were turned down for treatment solely based on CT or preoperative imaging results, and PTA was administered to patients with more than 50% central venous stenosis; however, if the patient's vascular elastic retraction was >50%, these patients were given sequential PTS. For patients with complete occlusion on central venography, sharp recanalization was performed by PTS. Angioplasty balloons were obtained from Boston Scientific (Boston, MA, USA). The balloon size was chosen based on the normal vascular diameter between the ends of the lesion. The endovascular stents used included the E-Luminexx bare metal stent (BARD, Murray Hill, NJ) and the G-V finger GORE® VIABAHN® covered stent (W.L. Gore & Associates, Flagstaff, AZ). The sharp recanalization technique can be used as an important supplement when the conventional blunt recanalization technique cannot cross the central vein occlusion (15–17). Sharp recanalization refers to the use of the stiff end of the guidewire or the sharp interventional instrument directly through the vascular occlusion segment and then into the vascular lumen on the other side of the occlusion segment. Commonly used sharp opening instruments include the stiff end of the guidewire, Chiba biopsy needle, transseptal needle, Rups-100 puncture needle, radiofrequency ablation guidewire, and trocar.

Technical failure is defined as the inability of the guidewire to pass through the lesion during the initial surgery. Technical success was defined as <30% residual stenosis and disappearance of abnormal collateral vessels around the phlebographic stenosis after endovascular treatment. Major complications are defined as diseases in which therapeutic intervention is performed within 30 days of surgery, including bleeding, hematoma, pneumothorax, hemothorax, mediastinal hematoma, and air embolism (18). Primary patency was defined as central venous patency without recurrent stenosis or need for further intervention. Adjunctive primary patency was defined as the central vein with further intervention to improve patency. All definitions were in accordance with the current standards of the Society for Vascular Surgery (SVS) and Society of Interventional Radiology (19).

### Follow-up and outcomes

Patient clinical data including technical failure, technical success, patency, and reasons for secondary procedures were recorded. Due to the difficulty of obtaining follow-up angiography data, clinical and/or hemodynamic measures of patency were assessed every 3 months for 12 months. Thus, central venous patency is clinically defined as the absence of limb swelling, pain, or access dysfunction. Follow-up clinical data were recorded from medical and hemodialysis databases.

### Statistical analysis

Normally distributed data are shown as the mean ± standard deviation. Non-normally distributed data were expressed as medians and interquartile ranges. Categorical data were expressed as percentages. Central venous patency rates were calculated using Kaplan-Meier analysis. The two groups were compared using the nonparametric Mann-Whitney *U*-test. Statistical significance was set than 0.05. Analyses were performed using SPSS software (version 26.0; IBM, New York, NY, USA).

## Results

### Patient characteristics

We enrolled 26 hemodialysis patients with CVS. There were 12 women and 14 men with a mean age of 54.96 ± 12.61 years (range: 30–77 years). The patients had been on hemodialysis for a mean of (6.08 ± 4.00) years (range 1–14 years). All patients had a history of central venous catheter or pacemaker implantation, and eight patients had combined fistula dysfunction. The clinical characteristics of the patients are summarized (Table 1).

**Table 1:** Characteristics of maintenance hemodialysis patients with central venous stenosis.

Item	*N* = 26
Gender	
Male	14 (53.85%)
Female	12 (46.15%)
Age (years)	54.96 ± 12.61
History of hemodialysis(years) history of catheter placement	6.08 ± 4.00
Internal jugular vein or subclavian vein	24（92.31%）
Single catheter placement	17
Multiple catheter placement	7
History of pacemaker implantation	2 (7.69%)
Duration of previous catheter retention (months) Primary disease	9（6–19.5）
Chronic glomerulonephritis	9 (34.61%)
Diabetic nephropathy	6 (23.08%)
Obstructive nephropathy	5 (19.23%)
Hypertensive nephropathy	3 (11.54%)
Others*	3 (11.54%)
Presenting symptom	
Limb edema	26 (100.00%)
Fistula dysfunction	8 (30.77%)

* Lupus nephritis, ANCA-associated vasculitis, and gout nephropathy.

### Treatment of central venous stenosis or occlusion

The patients were administered PTA alone or sequential PTS treatment based on the degree of stenosis and elastic retraction. A total of 26 patients underwent central venography; 7 patients showed complete occlusion of the central vein, and the remaining 19 patients were treated first with PTA, but only 9 patients achieved anatomical complete patency (residual stenosis <30%). Because PTA treatment alone did not meet the expected standards or the central vein was completely occluded, the other 17 patients were administered sequential PTS therapy. There was no difference in patient age, hemodialysis time, or catheter retention time between the sequential PTS and PTA alone groups. However, the degree of stenosis and proportion of occlusion in the sequential PTS group were more severe than those in the PTA alone group (Table 2).

**Table 2:** Comparison of clinical characteristics between PTS group and PTA group before treatment.

	PTS group	PTA group	*p*-value
Number of patients	17 (65.38)	9 (34.62)	—
Age, years	57.31 ± 2.93	54.57 ± 6.14	0.992
Male	7 (41.18)	7 (77.78)	0.081
Age of starting dialysis, years	51.92 ± 2.94	48.42 ± 6.69	0.978
Duration of HD, years	7.00 (2.00–9.00)	4.00 (2.00–13.00)	0.957
Stenosis degree, %	100.00 (95.00–100.00)	84.28 ± 6.40	0.001
Length of lesion, mm	30.77 ± 3.62	22.86 ± 5.10	0.199
Previous catheter retention time, months	12.00 (6.00–24.00)	6.00 (6.00–15.00)	0.558
Weight, Kg	55.35 ± 2.13	55.35 ± 2.31	0.809
SBP, mmHg	142.54 ± 7,51	136.71 ± 5.07	0.526
DBP, mmHg	86.15 ± 6.06	77.00 ± 2.60	0.159
MAP, mmHg	104.95 ± 6.36	96.91 ± 2.55	0.211
Pulse pressure, mmHg	56.38 ± 3.49	59.71 ± 5.45	0.872
HGB, g/L	105.15 ± 5.29	107.71 ± 6.16	0.424
WBC, 10^9/L	6.31 ± 0.55	6.86 ± 1.21	0.418
PLT, 10^9/L	229.00 ± 24.79	186.86 ± 18.74	0.377
BUN, mmol/L	20.73 ± 1.27	20.90 ± 3.31	0.632
Cr, *μ*mol/L	909.08 ± 73.33	783.69 ± 48.11	0.4
Ca, mmol/L	2.48 ± 0.08	2.39 ± 0.06	0.724
P, mmol/L	2.09 ± 0.15	2.23 ± 0.36	0.367
iPTH, pg/ml	485.66 ± 116.82	272.00 ± 96.43	0.284
LDH, U/L	210.46 ± 17.52	229.57 ± 18.46	0.588
ALB, g/L	40.20 ± 0.87	40.04 ± 2.04	0.846
UA, μmol/L	403.91 ± 33.50	338.00 ± 72.79	0.35
LDL-C, mmol/L	2.35 ± 0.19	2.41 ± 0.28	0.857
INR	1.02 ± 0.02	1.10 ± 0.03	0.016
FIB, g/L	3.99 ± 0.31	3.70 ± 0.29	0.537
D-Dimer, ng/ml	1,077.69 ± 208.78	1,084.29 ± 266.75	0.476
ALP, U/L	109.00 (72.50–139.00)	73.00 (64.00–90.00)	0.08
CRP, g/L	4.31 (1.28–10.38)	9.11 (3.08–16.03)	0.865
CK, U/L	76.00 (64.50–120.00)	110.00 (65.00–210.00)	0.62

In the sequential PTS group, venous sites were located in the subclavian vein (SCV) in of 7/17 patients (41.18%), brachiocephalic vein (BCV) in of 4/17 patients (23.53%), SVC (superior vena cava) in of 1/17 patients (5.88%), SCV + SVC in of 1/17 patients (5.88%), BCV + SVC in of 3/17 patients (17.65%), and SCV + BCV + SVC in of 1/17 patients (5.88%). In the PTA alone group, lesions venous sites were located in the brachiocephalic vein (BCV) in 8/9patients (23.53%) and SVC (superior vena cava (SVC) in 1/9 patients (5.88%). The incidence of brachiocephalic vein stenosis was significantly higher than that of subclavian vein stenosis (46.16% vs. 26.92%). The percentages of occlusion in the PTS and PTA alone groups were 11(11/17; 64.71%) and 1 (1/9; 11.11%), respectively. The differences between the two groups were statistically significant. The lesion venous sites and the rate of venous stenosis are summarized in Table 3.

**Table 3:** Lesions central venous sites and the rate of central venous stenosis.

Lesion sites	PTS group (*n* = 17)		PTA group (*n* = 9)		Total (*n* = 26)
	Type of lesions		Type of lesions		
	Stenosis	Occlusion	Stenosis	Occlusion	
SCV (subclavian vein)	2	5	0	0	7
BCV (brachiocephalic vein)	2	2	8	0	12
SVC (superior vena cava)	1	0	0	1	2
SCV + SVC	0	1	0	0	1
BCV + SVC	1	2	0	0	3
SCV + BCV + SVC	0	1	0	0	1
Total	6	11	8	1	26

SCV, subclavian vein; BCV, brachiocephalic vein; SVC, superior vena cava.

**Figure 1:**
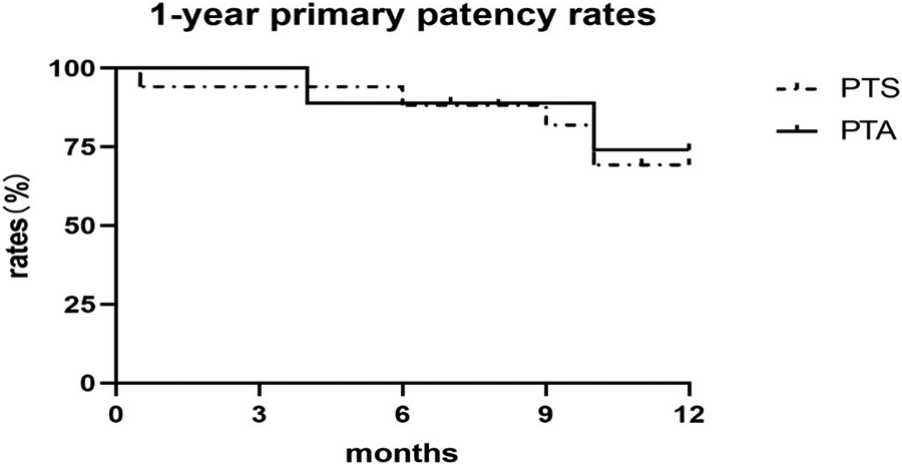
Primary patency of central vein stenosis after PTA or PTS. The PTA alone group is depicted with a solid line, and the sequential PTS group with a dashed line. Primary patency did not differ according to the Kaplan-Meier analysis (*P* = 0.823).

**Figure 2:**
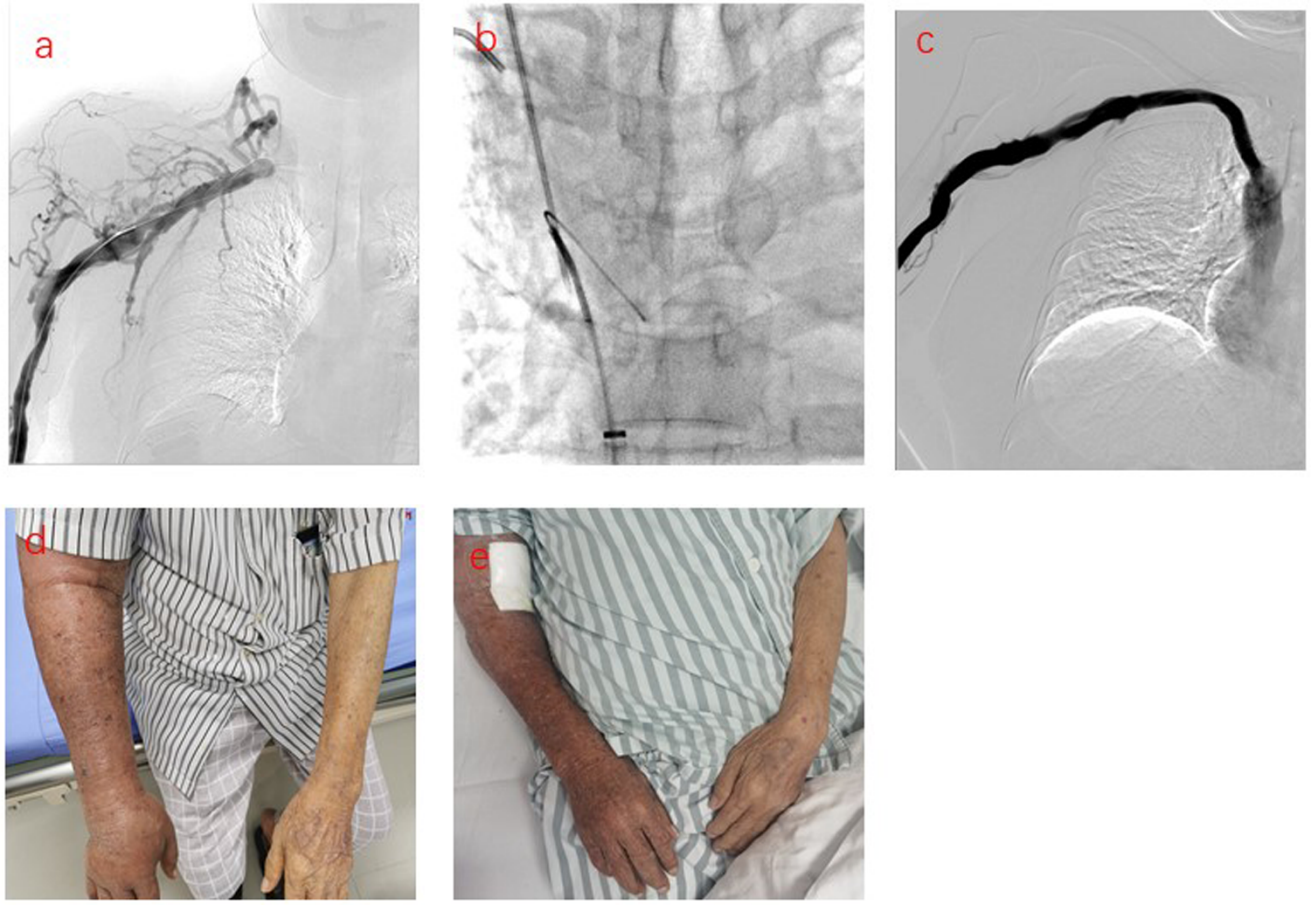
Sharp recanalization treatment for patients with central vein complete occlusion. **(a)** Phlebography showing occlusion of the right brachiocephalic vein. **(b)** The sharp needle entered the occluded right brachiocephalic vein, passed through the guidewire of the femoral vein, identified the occlusion site, and then the sharp needle punctured into the inferior vena cava, inserted the guidewire. **(c)** The stent was implanted into the right occluded brachiocephalic vein and the right brachiocephalic vein was restored to patency. d. The patient's right upper limb was swollen before treatment. **(e)** The swelling in the right upper extremity subsided after treatment.

The central venous primary patency rates in the sequential PTS and PTA alone groups were 94.12% and 100% at 3 months; 88.24% and 88.89% at 6 months; 75.00% and 85.71% at 9 months; and 66.67% and 71.43% at 12 months, respectively. There was no statistically significant difference between the sequential PTS and PTA alone group (Figure 1). In this study, 3 patients experienced technical failure. Although these cases did not result in successful interventions, they were included in our statistical analysis of failure rates. All 3 patients underwent secondary surgery during follow-up. PTA was successfully repeated in 2 patients, and vascular access was successfully reestablished in 1 patient at the contralateral limb. There were seven cases of complete occlusion of the brachiocephalic vein. The guidewire could not pass through the occlusion of the brachiocephalic vein. Sharp recanalization was performed, the stent was implanted into the blocked segment of the brachiocephalic vein, and the patients had successful penetration of the occlusion (Figure 2). The patency rates of sharp recanalization treatment for complete occlusion at 6 and 12 months were 85.71% and 71.43%, respectively. After PTA alone or sequential PTS, edema of the upper extremities and skin varicose veins significantly disappeared.

### Complications

No complications related to balloon angioplasty or vascular injury were observed. No complications, such as mediastinal hematoma, chest pain, thrombus, or vascular rupture, were observed in any patient. Severe adverse events such as acute pericardial effusion did not occur in these patients.

## Discussion

The accurate incidence of CVS in patients undergoing maintenance hemodialysis is unknown. The lesion is detected either as an incidental finding when a venogram is performed, or because the patient has typical signs or symptoms. If neither of these occurs, the lesion generally remains undetected. Recently, Al-Balas A, et al. (20). reported that the overall incidence of CVS in tunneled internal jugular venous central venous catheters was 13% in patients undergoing hemodialysis.

The most common cause of CVS is pacemaker or central venous catheter placement in patients (21). Although there was a significant increase in hemodialysis patients with arteriovenous fistula (AVF), most patients at the initiation of hemodialysis need to be placed on a non-tunneled catheter. Atieh AS et al. (4). reported that non-tunneled central venous catheters were used in 73% of hemodialysis patients, 13% with tunneled hemodialysis catheters, and 13% with AVF at hemodialysis initiation in the Palestinian hemodialysis center. Pisoni RL, et al. (5). reported that AVF use was 63% to 68% and catheter use was 15% to 19%. However, initiating hemodialysis patients with catheter was 70% in the United States. In our study, 26 patients underwent a total of 44 central venous catheter insertions, of which 39 were non-tunneled catheters and only 5 were tunneled catheters, for a total catheter indwelling time of 9 (6–19.5) months. In most hemodialysis patients, the signs and symptoms of CVS are mild or completely asymptomatic owing to collateral venous circulation. However, once an arterial venous fistula opens on the side of the stenosis of the central vein, collateral venous circulation may not be sufficient for venous drainage. High venous pressure can lead to edema of the arms, breasts, and/or face and even skin infections and ulcers in the upper extremities (22). Ideally, intervention when the vein stenosis is milder (e.g., about 50% stenosis) may result in a better prognosis, but due to the lack of early symptoms, this group of patients is often difficult to detect, often due to unexpected findings on angiography. In reality, patients often present with symptoms such as limb edema, when the stenosis is already high.

In this study, we successfully treated CVS in dialysis patients undergoing endovascular interventions. We found that all patients had a history of catheter placement or pacemaker implantation. According to the differences in treatment, the patients were divided into the PTA alone and sequential PTS groups. We compared the patency rates of PTA alone and sequential PTS treatment in hemodialysis patients with CVS. The primary patency rates in the PTS and PTA alone groups were 94.12% and 100% at 3 months; 88.24% and 88.89% at 6 months; 75.00% and 85.71% at 9 months; and 66.67% and 71.43% at 12 months, respectively. However, no statistically significant difference was observed between the sequential PTS and PTA alone groups. Our results show that PTA treatment alone with less than 50% central venous elastic retraction is consistent with sequential PTS patency in patients with central venous elastic retraction more than 50%.

One-year primary patency after PTA alone has been reported to be between 20% and 50% in the literature (23–25). Our study results showed a higher patency rate at one year than that reported in previous studies. The present study showed similar primary patency outcomes in the sequential PTS and PTA alone groups. However, sequential PTS is a salvation treatment after PTA treatment failure. Although stenting may have superior patency, there are some disadvantages in stent placement, such as the possibility of endovascular hyperplasia, and the number of re-interventions after stenting may be higher than that with PTA alone therapy.

It is worth noting that for 7 patients with complete occlusion, we used sharp recanalization technology and stenting placement, with patency rates of 85.71% and 71.43% at 6 and 12 months, respectively. Sharp recanalization can increase the technical success rate of central vein occlusion treatments. Sharp recanalization is an important remediation technique when the conventional blunt recanalization technique cannot cross the central vein occlusion. Sharp recanalization refers to the use of sharp interventional instruments directly through the vascular occlusion segment and then into the vascular lumen on the other side of the occlusion segment. Before sharp recanalization, enhancement computed tomography (CT), magnetic resonance imaging (MRI), or digital subtraction angiography (DSA) should be performed to determine the position, direction, length, diameter, and other conditions of the vascular occlusion segment, which is helpful in selecting the appropriate surgical approach, surgical instruments, opening direction, and length of opening puncture. A dilated balloon or stent must be placed when the guidewire passes through the occlusive lesion (17). In the process of sharp recanalization, we need to pay more attention should be paid to severe complications such as mediastinal hematoma, hemothorax, and pericardial effusion.

This study had some limitations. First, this was a retrospective study, and randomized controlled trials may be more useful in evaluating the effective treatment of sequential PTS or PTA alone in hemodialysis patients with CVS. Second, this is a study with a small sample size, and we need to accumulate more samples to analyze the efficacy of sequential PTS and PTA treatment alone. Third, catheter and pacemaker implantation history are both high risk factors for central vein stenosis in hemodialysis patients. Although the mainstream view is that it is related to endothelial proliferation caused by physical stimulation, the specific mechanism is still unknown. A total of 3 patients had a history of pacemaker placement, and these 3 patients also had a history of catheter placement. Only one person whose central vein stenosis could be considered to be caused by a pacemaker (judging from the location of his catheter) had a stent implanted, while the other two, one received PTA and the other received stent implantation. Two patients who underwent stenting had access patency at the end of the observation period, while patients who underwent PTA experienced technical failure, re-failure of access, and contralateral re-access. Due to the difficulty in identifying the culprits of central vein stenosis and the large difference between the pacemaker group and the catheter group, it is difficult to compare the patency rates of the two groups. Finally, all patients were started on PTA therapy, and when PTA treatment failed, PTS was selected as the remedial treatment for PTA. Patients who received sequential PTS differed from those who received PTA. However, our findings suggest that PTA treatment alone with less than 50% central venous elastic retraction is consistent with sequential PTS patency in patients with central venous elastic retraction ≥ 50%.

## Conclusions

Maintenance hemodialysis patients with CVS often have a history of catheter placement or pacemaker implantation. PTA alone is recommended for patients with less than 50% central venous elastic retraction, while sequential PTS is recommended for patients with ≥50% central venous elastic retraction. PTA and PTS are safe and effective methods for the treatment of CVS in patients undergoing hemodialysis.

## Data Availability

The datasets presented in this study can be found in online repositories. The names of the repository/repositories and accession number(s) can be found in the article/Supplementary Material.
